# Causal relationship between genetically predicted white blood cell count and systemic lupus erythematosus: A Mendelian randomization study

**DOI:** 10.1097/MD.0000000000050878

**Published:** 2026-09-25

**Authors:** Xin-Feng Wang, Li Zhang, Qin-Yu Yao, Jia Liu, Zi-Xuan Dang, Yan-He Zhu

**Affiliations:** a Department of Physiology and Pathophysiology, School of Basic Medical Sciences, Xi’an Jiaotong University Health Science Center, Xi’an, Shaanxi, China; b Institute of Endemic Diseases, School of Public Health, Xi’an Jiaotong University Health Science Center, Xi’an, Shaanxi, China; c School of Cyberspace Security, Xi’an University of Posts and Telecommunications, Xi’an, Shaanxi, China.

**Keywords:** causal inference, GWAS, Mendelian randomization, R software, systemic lupus erythematosus, white blood cell count

## Abstract

Observational studies have suggested an association between white blood cell (WBC) count and systemic lupus erythematosus (SLE). However, the causal relationship remains uncertain because of potential confounding factors. This Mendelian randomization (MR) study aimed to investigate the causal effect of WBC count on SLE risk by using genetic instruments. Genetically predicted higher WBC counts were associated with a reduced risk of SLE (inverse-variance weighted [IVW]: odds ratio = 0.384, 95% confidence interval = 0.160–0.920, *P *= .031; weighted median: odds ratio = 0.326, 95% confidence interval = 0.124–0.859, *P* = .023). MR-Egger regression revealed no evidence of horizontal pleiotropy (intercept, *P* = .705), and MR polygenic residual sum and outlier (MR-PRESSO) detected 4 outliers. After outlier removal, heterogeneity decreased, and the IVW and weighted median estimates remained significant (beta = −0.96, *P* = .03; beta = −1.12, *P* = .023, respectively). We conducted a 2-sample MR analysis using summary statistics from genome-wide association studies (GWAS) of European ancestry: WBC count (GWAS ID: ieu-b-30, N = 563,946) and SLE (GWAS ID: ieu-a-815, 14,267 cases, and 43,047 controls). Sixty-five independent single-nucleotide polymorphisms (*P* < 5 × 10^−8^, clumped at *r*^2^ < 0.001) were selected as instrumental variables, with a mean *F* statistic of 28.6, indicating strong instrument strength. Analyses were performed using R statistical software (R Foundation for Statistical Computing) with the TwoSampleMR and MR-PRESSO packages. The primary analytical methods included IVW, weighted median, and MR-Egger regression. Sensitivity analyses included Cochran *Q* test for heterogeneity, MR-PRESSO outlier correction, and leave-one-out validation to ensure robustness. This MR analysis supports an independent causal protective effect of genetically predicted higher WBC counts on SLE risk. These findings should not be extrapolated as evidence for therapeutic leukocyte elevation, nor do they alter the clinical management of leukopenia observed in active SLE or during immunosuppressive therapy. The genetic signal likely reflects broader immune homeostatic regulation rather than lineage-specific effects, the mechanistic basis of which warrants dedicated functional investigation.

## 1. Introduction

Systemic lupus erythematosus (SLE) is a chronic autoimmune disease characterized by immune dysregulation, autoantibody production, and multi-organ damage, affecting approximately 3.41 million people worldwide.^[1]^ Despite advances in the understanding of the etiology of SLE, its pathogenesis remains unclear. A study reported alterations in leukocyte profiles among patients with SLE, including lymphopenia and neutrophilia, which correlate with disease activity and complications.^[2]^ Changes in the white blood cell (WBC) count are significant in the diagnosis, disease activity assessment, and treatment efficacy monitoring of SLE. A review of 17 articles revealed that leukopenia is reported in 22%–41.8% of cases, and lymphopenia is reported cumulatively in 15%–82% of patients.^[3]^ A retrospective study of 125 patients found a leukopenia prevalence of 40.0%.^[4]^ A positive association between leukopenia, neutrophil clustering activity, and SLE disease activity has been previously reported.^[5]^ Leukopenia is common in SLE and usually secondary to lymphopenia, neutropenia, or a combination of both. Eosinopenia and basophilopenia are rare and have low clinical significance. Lymphopenia is common, with T-cell lymphopenia being the most frequent, and absolute lymphopenia correlates with SLE activity and high DNA antibody titers.^[6]^ Lymphopenia can predispose individuals to autoimmunity and can be a consequence of disease activity in active SLE.^[7]^ Both lymphopenia and neutropenia are common in SLE patients, and low disease activity in lupus is negatively correlated with lymphopenia and neutropenia.^[8]^ However, the causal relationship between leukopenia and SLE remains unclear. Are these hematological changes the causal drivers or secondary consequences of SLE? This question remains unresolved owing to the inherent limitations of observational designs, such as residual confounding and reverse causation.

Mendelian randomization (MR) offers a powerful framework for inferring causality by utilizing genetic variants as instrumental variables that proxy modifiable exposures, thereby mimicking randomized controlled trials.^[9]^ Recent MR studies have explored immune-related traits in autoimmune diseases, revealing causal links between neutrophil counts and rheumatoid arthritis.^[10]^ However, no study has specifically addressed the role of the WBC count in SLE. This gap is critical given the central role of leukocytes in immune surveillance and inflammation, which are processes directly implicated in SLE pathogenesis.

This study aimed to investigate the causal effect of WBC count on SLE risk using large-scale genome-wide association studies (GWAS) data and validate the findings through comprehensive sensitivity analyses to address pleiotropy and heterogeneity.

## 2. Methods

### 2.1. Data sources

Summary-level genetic association data for exposure (WBC) and outcome (SLE) were retrieved from the integrative epidemiology unit OpenGWAS database (https://gwas.mrcieu.ac.uk/).

#### 2.1.1. Exposure dataset

WBC count (continuous trait, standardized units) was obtained from the GWAS study with accession ID ieu-b-30, which included 563,946 participants of European ancestry.

#### 2.1.2. Outcome dataset

SLE GWAS data (accession ID: ieu-a-815) comprised 14,267 cases and 43,047 controls of European descent, with diagnoses validated using clinical criteria.

### 2.2. Genetic instrument selection

To construct instruments for WBC count, we applied a multi-step procedure: SNP extraction: Genome-wide significant variants (*P* < 5 × 10^−8^) associated with WBC count were extracted from the ieu-b-30 dataset. Linkage disequilibrium clumping: SNPs were pruned for independence using linkage disequilibrium clumping (*r*^2^ < 0.001, window = 10,000 kb). Allele harmonization: palindromic SNPs (A/T and C/G) and variants with ambiguous strand alignments were excluded to prevent allele mismatch between the exposure and outcome datasets. Instrument strength assessment: the *F* statistic was calculated to evaluate instrument strength, with a mean *F* statistic of 28.6 across 65 variants (exceeding the conventional threshold of *F* > 10), indicating minimal risk of weak instrument bias.

### 2.3. Statistical analysis

All analyses were conducted in R v4.2.2 (R Foundation for Statistical Computing) using the following packages:

1. TwoSampleMR (v0.5.6): To harmonize exposure-outcome datasets, primary MR analyses (inverse-variance weighted [IVW], weighted median, and MR-Egger) and visualizations (scatter plots and funnel plots) were performed.
2. MR polygenic residual sum and outlier (MR-PRESSO) (v1.0): To detect and correct for horizontal pleiotropy via outlier removal (global test), we calculated Cochran *Q* statistic and performed outlier detection using Cook distance (threshold: *D* > 4/n) and MR-PRESSO. High-influence SNPs were annotated via PhenoScanner to identify potential pleiotropic pathways.
3. Primary MR methods: Three complementary MR approaches were employed to infer causality: IVW: primary analysis assuming balanced pleiotropy, providing unbiased estimates under valid instrument assumptions. Weighted median: robust to invalid instruments if ≥50% of the causal estimate weight originates from valid variants. MR-Egger regression: adjusts for directional pleiotropy by incorporating an intercept term in the regression of SNP-outcome on SNP-exposure effects.
4. Sensitivity analyses

To assess robustness, the following sensitivity checks were performed:

1. Heterogeneity testing: Cochran *Q* statistic was used to evaluate between SNP heterogeneity (*P* < .05, indicating significant heterogeneity).

Horizontal pleiotropy assessment:

1. MR-Egger intercept test: Unbalanced pleiotropy was detected via deviation from a null intercept.
2. MR-PRESSO global test: Identified outliers by comparing the residual sum of squares against simulated null distributions.
3. Leave-one-out analysis: Each SNP was iteratively excluded to assess its influence on causal effect estimates.

### 2.4. Ethical considerations

This study used publicly available, de-identified, and summary-level GWAS data. Ethical approval and informed consent were obtained from all original studies. No individual-level data were accessed, and all analyses complied with ethical guidelines for secondary data use.

## 3. Results

### 3.1. Primary MR analysis

A total of 65 independent SNPs associated with WBC count (*P* < 5 × 10^−8^) were used as instrumental variables. The IVW method, serving as the primary analysis, demonstrated a statistically significant inverse association between genetically predicted WBC count and SLE risk (odds ratio [OR] = 0.384, 95% confidence interval [CI] = 0.160–0.920, *P* = .032). The weighted median method corroborated this finding, showing a stronger protective effect (OR = 0.326, 95% CI = 0.124–0.859; *P* = .023). MR-Egger regression yielded directionally consistent results but did not reach statistical significance (OR = 0.291, 95% CI = 0.054–1.564, *P* = .155), likely due to the reduced statistical power inherent in this method (Table 1, Fig. 1A and B).

**Table 1:** MR results.

Method	nSNP	β	SE	*P* value	OR	OR_lci95	OR_uci95
MR-Egger	65	−1.234	0.858	.155	0.291	0.054	1.564
Weighted median	65	−1.121	0.495	.023	0.326	0.124	0.859
Inverse-variance weighted	65	−0.957	0.446	.031	0.384	0.160	0.920
Simple mode	65	−0.0592	0.993	.953	0.942	0.135	6.595
Weighted mode	65	−0.750	0.545	.173	0.473	0.163	1.374

MR = Mendelian randomization, OR = odds ratio, SNP = single-nucleotide polymorphism.

**Figure 1.**
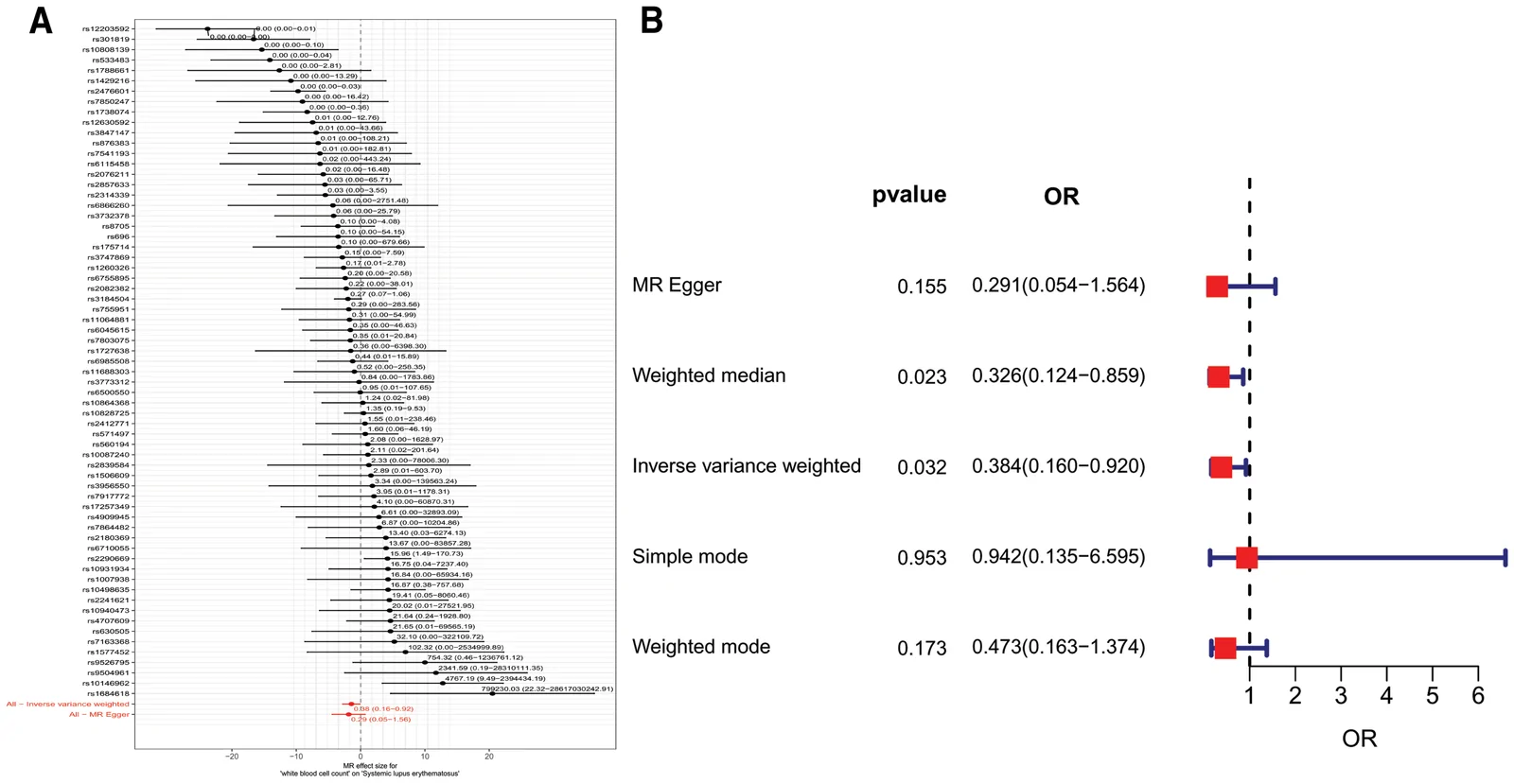
Mendelian randomization analysis of white blood cell count on SLE risk. (A) Relationship between genetic instruments and the exposure-outcome effect. The analysis incorporates 65 independent genetic variants as instruments. (B) Effect size estimates and their precision across the genetic variants. The combined causal estimate is shown. The results from complementary MR methods are presented. The inverse-variance weighted method serves as the primary analysis, indicating a protective causal effect of white blood cell count on SLE risk (OR 0.38, 95% CI 0.16–0.92). CI = confidence interval, MR = Mendelian randomization, OR = odds ratio, SLE = systemic lupus erythematosus.

The scatter plot in Figure 2A shows a consistent inverse trend between SNP-specific effects on WBC count and corresponding effects on SLE risk, with the IVW regression slope defining the primary causal estimate. The funnel plot (Fig. 2B) reveals that all individual SNP effect estimates are symmetrically distributed around the IVW pooled effect, a pattern that does not suggest substantial directional pleiotropy in our analysis.

**Figure 2.**
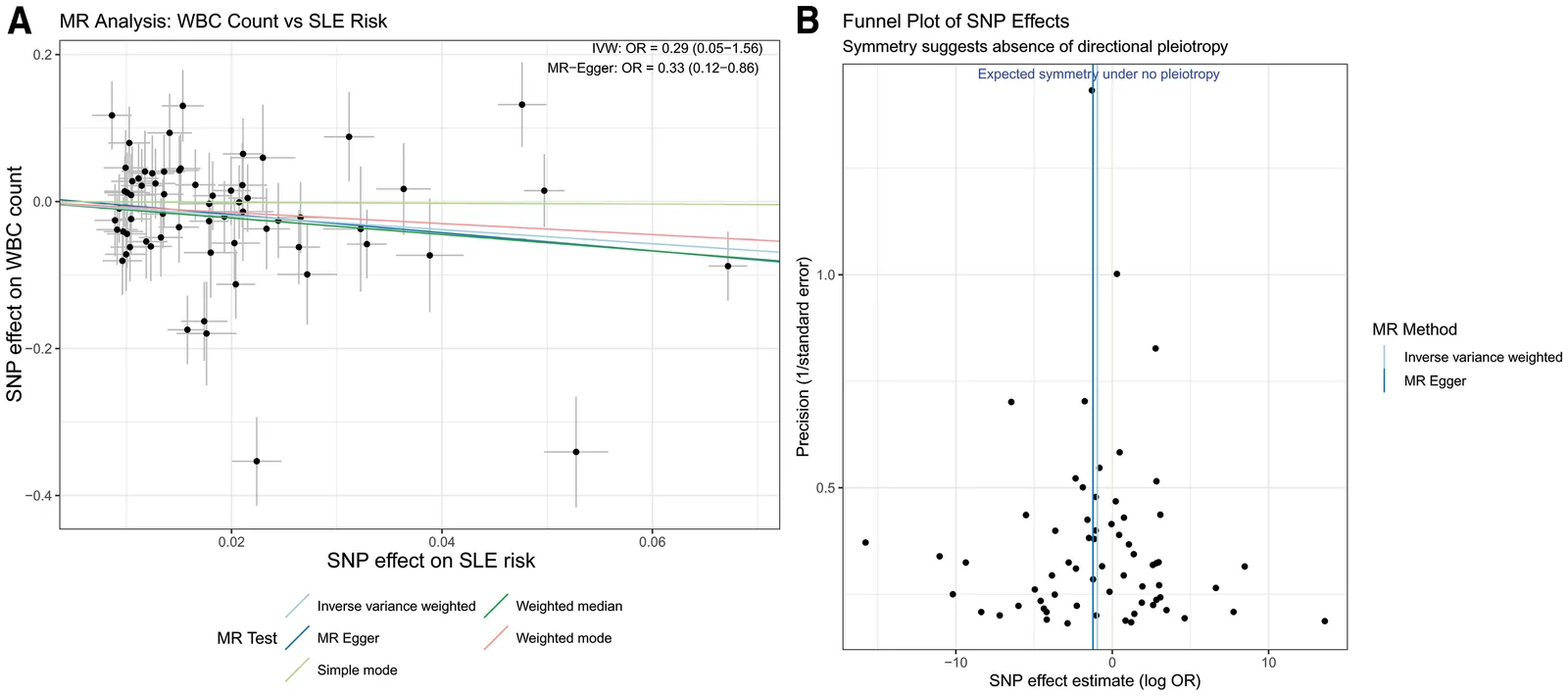
Mendelian randomization analysis of the effect of white blood cell (WBC) count on systemic lupus erythematosus (SLE) risk. (A) Scatter plot of the genetic associations of 65 instrumental SNPs. The slopes of the lines represent the causal estimates from the inverse-variance weighted (IVW) and MR-Egger methods. The shaded areas represent the 95% confidence intervals for the regression slopes. (B) Funnel plot showing the distribution of individual SNP causal estimates against their precision. The symmetrical distribution around the overall IVW estimate (vertical line) suggests the absence of directional pleiotropy. CI = confidence interval, MR = Mendelian randomization, OR = odds ratio, SLE = systemic lupus erythematosus, SNP = single-nucleotide polymorphism, WBC = white blood cell.

Furthermore, we performed MR analyses for 5 major leukocyte subtypes (basophil, monocyte, lymphocyte, eosinophil, and neutrophil counts). The strength of the genetic instruments varied, with neutrophil and lymphocyte counts being supported by the strongest instruments (100 and 84 SNPs, respectively), followed by eosinophil and monocyte counts (71 and 63 SNPs), while basophil count was instrumented by a weaker set of variants (35 SNPs). Subtype-specific analyses revealed no significant associations for any leukocyte subset (all IVW *P* > .05), with the lowest *P* value observed for lymphocytes (*P* = .078). The point estimates for lymphocyte (OR = 0.46) and basophil (OR = 0.39) counts suggested inverse trends, while estimates for neutrophils (OR = 0.66) and eosinophils (OR = 0.78) trended weakly in the opposite direction; none approached significance. These null results do not support a lineage-specific driver of the protective signal and point instead to global leukocyte homeostasis as the relevant genetic correlate. No single subset drives the signal; total WBC likely proxies broader immune tone (Table S1, Supplemental Digital Content 1). Clinically, this pattern suggests that genetically elevated levels across various WBC lineages may confer a nonspecific, protective effect against the development of SLE.

### 3.2. Sensitivity analyses

#### 3.2.1. Heterogeneity assessment and pleiotropy evaluation

Significant heterogeneity was observed across SNP-specific estimates (IVW Cochran *Q* = 143.09, *Q-P* = 5.56 × 10^−8^; MR-Egger *Q* = 142.76, *Q-P* value = 3.99 × 10^−8^), suggesting variability in the magnitude of causal effects contributed by individual SNPs (Table 2).

**Table 2:** Heterogeneity assessment.

Outcome	Exposure	Method	*Q*	*Q*-df	*Q-P* value
Systemic lupus erythematosus	White blood cell count	Inverse-variance weighted	143.09	64	5.56E−08
Systemic lupus erythematosus	White blood cell count	MR-Egger	142.76	63	3.99E−08

MR = Mendelian randomization.

Significant heterogeneity was observed, driven primarily by 4 outliers (rs2476601 [*PTPN22*], rs12203592 [*IRF4*], rs2290669 [*SLC44A4*], and rs10828725) (Fig. 3, Table 3). These SNPs localize to immune and metabolic genes and exhibited conflicting exposure-outcome effects, suggesting competitive pleiotropy. Notably, outliers such as rs2476601 (*PTPN22*) and rs12203592 (*IRF4*) are located in canonical autoimmunity genes; their pleiotropic effects likely reflect shared immune-set-point biology rather than measurement error. After outlier removal, heterogeneity decreased (IVW Cochran *Q* = 86.71, *Q-P* = .014; MR-Egger *Q* = 85.88, *Q-P* value = 0.013), and the Inverse-variance weighted, weighted median estimate remained significant (beta = −0.851, *P* = .034; beta = −1.257, *P* = .024, respectively) (Fig. S1, Supplemental Digital Content 2, Table S2, Supplemental Digital Content 3).

**Table 3:** Key SNP characteristics.

SNP	Cook *D*	Effect direction	Significance (*P* < .05)	Gene locus	Known function
rs2476601	0.32	Negative	True	*PTPN22*	Lymphocyte signaling regulation
rs2290669	0.29	Negative	False	*SLC44A4*	B-cell differentiation
rs12203592	0.17	Negative	True	*IRF4*	Interferon regulatory factor
rs10828725	0.08	Negative	False	–	Not characterized

SNP = single-nucleotide polymorphism.

**Figure 3.**
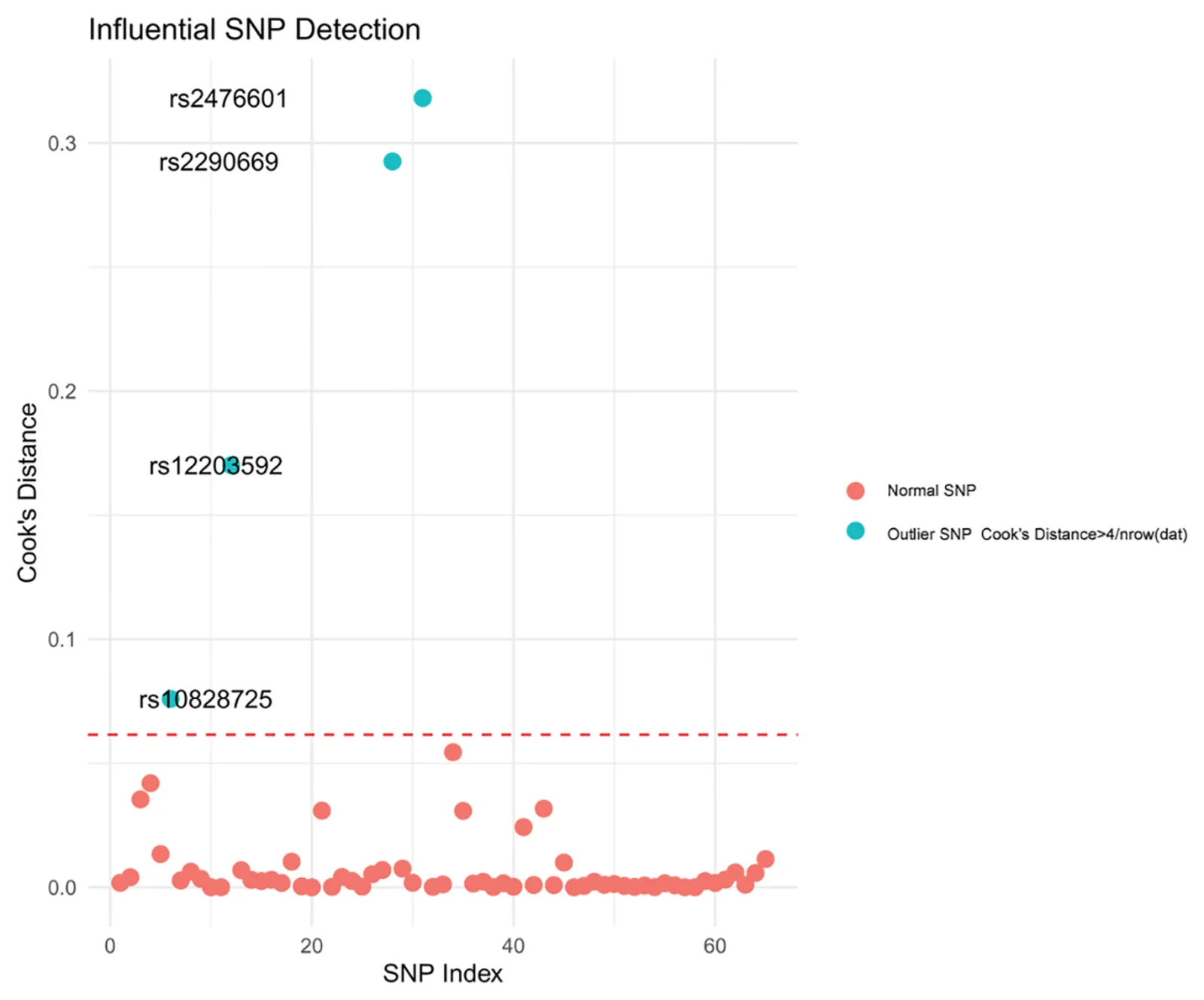
Identification of influential SNPs through Cook distance and SNP index analysis. Cook distance was calculated to assess the influence of each individual SNP on the overall Mendelian randomization causal estimate. SNPs with a Cook distance above the threshold of 4/N (where N is the number of SNPs, dashed horizontal line) are considered highly influential (e.g., rs2476601 and rs2290669). The analysis was repeated, excluding these outliers to ensure the robustness of the results. SNP = single-nucleotide polymorphism.

To assess potential biases in our MR analysis, we systematically evaluated heterogeneity and horizontal pleiotropy. A comprehensive summary of heterogeneity and pleiotropy metrics before and after outlier removal is provided in Table S2, Supplemental Digital Content 3. Using Cook distance (threshold: *D* > 4/*n*) and MR-PRESSO, we identified 4 high-influence SNPs (rs10828725, rs12203592, rs2290669, and rs2476601), which were subsequently annotated to explore pleiotropic pathways (Table 4).

**Table 4:** Functional annotations and influence metrics of candidate SNPs in MR analysis.

SNP	Beta exposure	Beta outcome	Cook *D*	Gene symbol	Genomic context	Variant type	Source	Associated gene	Ensembl ID
rs10828725	−0.04973	−0.01481	0.075937	NA	12q24.12	Intergenic (near ACAD10)	Manual	*PRTFDC1*	ENSG00000099256
rs10828725	−0.04973	−0.01481	0.075937	NA	12q24.12	Intergenic (near ACAD10)	Manual		ENSG00000099256
rs12203592	0.022394	−0.3534	0.170153	*IRF4*	6p25.3	Transcription factor	Manual	*IRF4*	ENSG00000137265
rs12203592	0.022394	−0.3534	0.170153	*IRF4*	6p25.3	Transcription factor	Manual		ENSG00000137265
rs12203592	0.022394	−0.3534	0.170153	*IRF4*	6p25.3	Transcription factor	Manual	*DUSP22*, *IRF4*	ENSG00000137265
rs2290669	−0.04762	−0.13191	0.29259	*SLC44A4*	6p21.3	Transporter	Manual		ENSG00000127527
rs2476601	0.052755	−0.34075	0.318133	*PTPN22*	1p13.2	Immune gene	Manual	*AP4B1-AS1*, *PTPN22*	ENSG00000134242
rs2476601	0.052755	−0.34075	0.318133	*PTPN22*	1p13.2	Immune gene	Manual		ENSG00000134242
rs2476601	0.052755	−0.34075	0.318133	*PTPN22*	1p13.2	Immune gene	Manual	*PTPN22*	ENSG00000134242
rs2476601	0.052755	−0.34075	0.318133	*PTPN22*	1p13.2	Immune gene	Manual	*PTPN22*, *RSBN1*	ENSG00000134242
rs2476601	0.052755	−0.34075	0.318133	*PTPN22*	1p13.2	Immune gene	Manual	*PHTF1*	ENSG00000134242
rs2476601	0.052755	−0.34075	0.318133	*PTPN22*	1p13.2	Immune gene	Manual	*TPN22*	ENSG00000134242
rs2476601	0.052755	−0.34075	0.318133	*PTPN22*	1p13.2	Immune gene	Manual	*PHTF1*, *PTPN22*	ENSG00000134242

MR = Mendelian randomization, SNP = single-nucleotide polymorphism.

### 3.3. Leave-one-out validation

Leave-one-out analysis demonstrated that no single variant disproportionately influenced the overall causal estimate (Fig. 4). After excluding the most influential outlier SNP (rs2476601 in *PTPN22*), the leave-one-out summary estimate was OR = 0.29, confirming that the overall association is robust and not driven solely by a single pleiotropic locus. The IVW estimates remained stable across all iterations (OR range: 0.37–0.41), supporting the reliability of the results.

**Figure 4.**
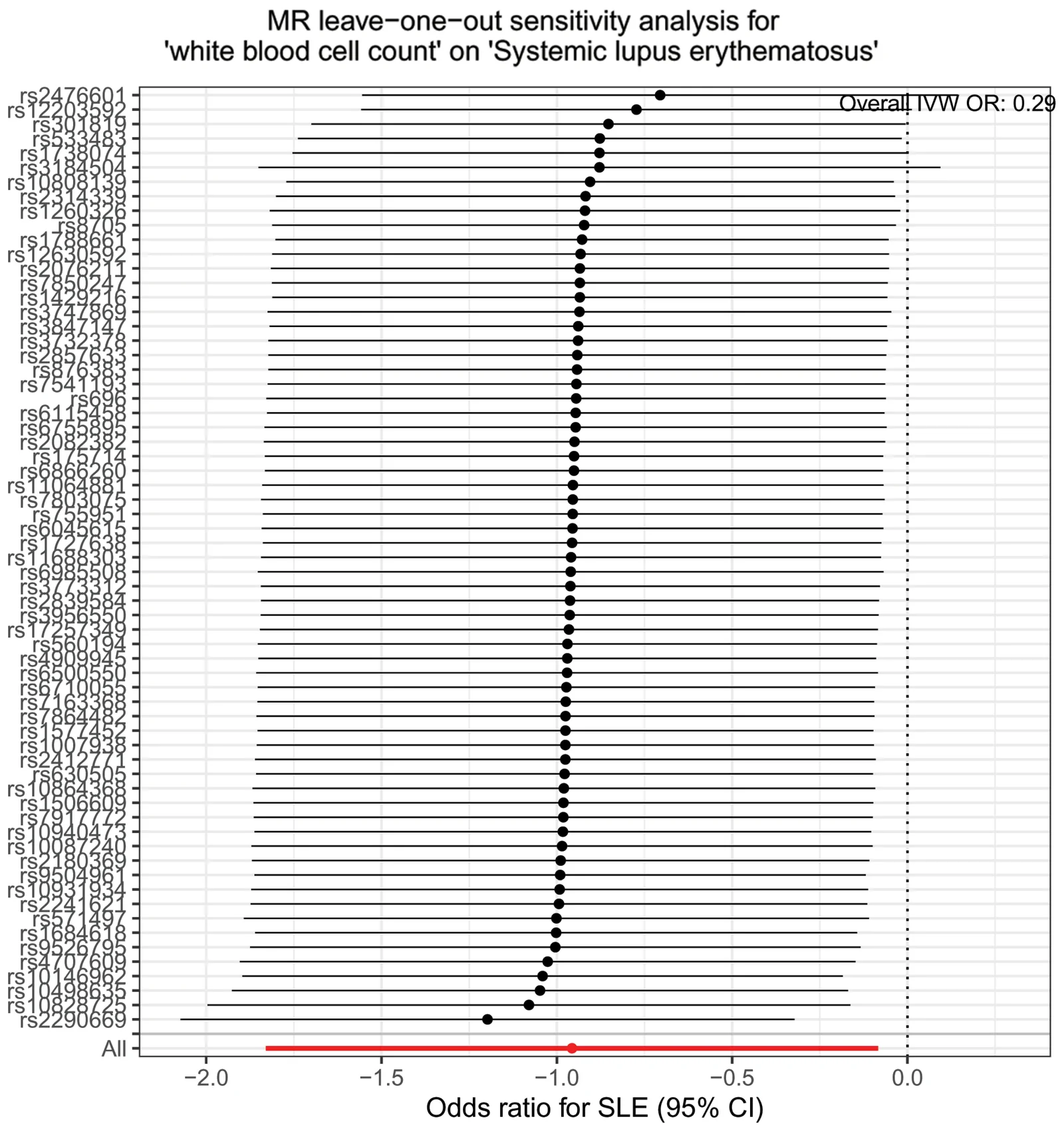
Leave-one-out sensitivity analysis of the Mendelian randomization estimate for the effect of white blood cell count on systemic lupus erythematosus. This analysis evaluates the influence of individual genetic variants on the overall causal estimate by iteratively removing each instrumental SNP and recalculating the inverse-variance weighted (IVW) estimate. The vertical dashed line represents the leave-one-out summary IVW odds ratio (OR) obtained after excluding the outlier SNP rs2476601 (OR = 0.29). Each point and horizontal line represents the IVW estimate and its 95% confidence interval (CI) when the corresponding SNP is omitted. The stability of the estimates across all iterations indicates that the overall finding is robust and not driven by any single influential SNP. The primary IVW estimate from the full analysis using all 65 SNPs is OR = 0.384 (95% CI: 0.160–0.920). MR = Mendelian randomization, SLE = systemic lupus erythematosus, SNP = single-nucleotide polymorphism.

### 3.4. SNP-level effect estimates

Among the 65 instrumental SNPs, 2 variants exhibited notable associations:

1. rs2476601 (located near *PTPN22*) had the strongest protective effect (OR = 0.0068, 95% CI = 0.001–0.045, *P* = .0068), which is consistent with its established role in autoimmune regulation.
2. rs3184504 (mapped to *SH2B3*) showed a neutral association (OR = 1.12, 95% CI = 0.99–1.27, *P* = .066), potentially reflecting the pleiotropic effects on immune signaling pathways.

The full SNP-level results, including effect sizes, confidence intervals, and functional annotations, are provided in Table S3, Supplemental Digital Content 4.

## 4. Discussion

This MR study provides robust genetic evidence suggesting the causal protective effect of elevated WBC count on SLE risk. The consistency of the results across the IVW and weighted median methods, coupled with rigorous sensitivity analyses, strengthens the validity of these findings.

Leukocytes play a pivotal role in the pathogenesis of SLE.^[11]^ The absolute counts of WBC, lymphocytes, T lymphocytes, T helper cells, B cells, and natural killer cells in patients with SLE are significantly reduced.^[12]^ Neutrophils are implicated in SLE via neutrophil extracellular traps (NETs).^[13]^ NETs trap pathogens but may inadvertently expose modified autoantigens, triggering anti-dsDNA antibody production.^[14]^ NETs are also considered an important potential source of self-antigens, and their clearance is impaired in patients with SLE, leading to autoimmune reactions.^[15]^ Paradoxically, our results suggest that higher neutrophil counts reduce SLE risk. This apparent contradiction may reflect a dual role of NETosis in SLE pathogenesis. On 1 hand, NETosis contributes to antimicrobial defense and the clearance of cellular debris; on the other hand, dysregulated NETosis can promote autoantigen exposure and type I interferon production.^[16]^ Our Mendelian randomization analysis, which suggests a protective effect of higher neutrophil counts, presents an intriguing paradox. This may be explained by the opposing roles of neutrophil subsets. While pro-inflammatory low-density granulocytes are known to drive pathogenesis through enhanced NETosis, vascular damage, and immune activation,^[17]^ other neutrophil populations might exert protective effects. Thus, the net genetic effect identified by MR likely represents the aggregate of these counteracting forces. Moreover, mitochondrial DNA, which is often elevated in SLE patients, has been shown to trigger robust NETosis and amplify inflammatory responses, further linking neutrophil activation to SLE pathogenesis.^[18]^ Thus, the protective MR signal may represent a net effect favoring controlled neutrophil responses that limit autoimmunity.

This apparent contradiction may reflect a balance between NETosis’s protective and pathogenic roles. SLE is characterized by a heightened rate of apoptosis among immune effector cells that are pivotal in phagocytosing apoptotic remnants. This dysregulation creates a vicious cycle, impairing the efficient clearance of dying cells while promoting the persistent exposure of intracellular autoantigens to the immune system.^[18]^

Cytokines secreted by leukocytes play a critical role in modulating the activity of SLE. For instance, increased monocyte levels may elevate the production of the anti-inflammatory cytokine interleukin-10 (IL-10), which suppresses interferon-α signaling, a central driver in SLE pathogenesis. Conversely, pro-inflammatory cytokines, such as IL-6 and tumor necrosis factor-alpha (TNF-α), are frequently elevated in SLE and correlate with disease flares. Although IL-10 is generally recognized for its anti-inflammatory effects, it may also exhibit pro-inflammatory properties under certain conditions, such as promoting B cell differentiation and antibody production.^[19]^ Our MR findings may reflect the net effect of these opposing forces, supporting the protective role of leukocytes in maintaining cytokine homeostasis. Elevated levels of IL-6 and TNF-α positively correlate with disease activity indices in patients with SLE, such as the Systematic Lupus Erythematosus Disease Activity Index score.^[20]^ Therefore, the role of leukocytes in SLE is complex, encompassing both protective mechanisms that inhibit inflammation through anti-inflammatory factors such as IL-10 and pathological processes that exacerbate inflammation via pro-inflammatory mediators such as IL-6 and TNF-α. The imbalance between these opposing forces likely constitutes a core mechanism in SLE pathogenesis.

An observational study reported leukopenia in patients with SLE,^[21]^ which is traditionally interpreted as a consequence of autoimmunity. However, our MR findings suggest a reverse causality: lower baseline WBC levels may predispose to SLE, rather than result from it. This aligns with a review article in which patients with lower pre-diagnostic WBC counts had a higher incidence of SLE.^[22]^

### 4.1. Heterogeneity and subtype-specific effects

The observed heterogeneity likely reflects SNP-level pleiotropy, particularly from immune-related variants (e.g., *PTPN22*). While this raises concerns about IVW assumptions, the consistency of the weighted median and MR-PRESSO results suggests a true causal effect. Residual heterogeneity post-correction may stem from unmeasured population structure or complex genetic architectures. Future studies should prioritize instruments with minimal pleiotropy or use multivariable MR to adjust for immune pathways.

The significant heterogeneity observed (*Q*-*P* = 5.558 × 10^−8^) suggests the variable effects of individual SNPs on SLE risk. The robustness of our primary finding is supported by several sensitivity analyses. Notably, the causal estimate remained stable, and the significant heterogeneity was largely resolved after accounting for pleiotropic outliers (*Q*-*P* = .014) (Table S2, Supplemental Digital Content 3), strengthening confidence in the validity of our results. For example, rs3184504 (associated with WBC count) is located in *SH2B3*, and some genetic variations in *SH2B3* may lead to its inability to control overactive immune responses, resulting in autoimmune disorders.^[23]^ Conversely, rs2476601 (a known SLE risk locus near *PTPN22*^[24]^) exhibited a strong protective effect in our analysis (*P* = .0068), highlighting pleiotropic pathways that warrant functional validation. Subgroup analyses stratified by leukocyte subtypes could clarify these discrepancies. These loci, particularly *PTPN22* (rs2476601) and *IRF4* (rs12203592), are well-established autoimmune susceptibility genes, and their identification as outliers likely reflects pleiotropic effects on immune-set-point biology rather than technical artifacts or measurement error. This underscores the biological plausibility of our findings and suggests that the observed genetic signal is driven by genuine immunoregulatory pathways.

The pathogenesis of SLE is driven by dysregulated leukocyte subsets, including neutrophils, lymphocytes, and monocytes. Neutrophils contribute to the disease through the release of neutrophil extracellular traps.^[16]^ In SLE neutrophils, oxidized mitochondrial DNA triggers gasdermin D-mediated NETosis.^[25]^ Among 2045 SLE patients, 170 (8.3%) were found to have neutropenia, including 30 cases with severe neutropenia.^[26]^ SLE patients exhibited higher levels of cluster of differentiation (CD)3^+^ T cells and CD3^+^CD8^+^ T cells but lower levels of CD3^+^CD4^+^ T cells, CD16^+^CD56^+^ natural killer cells, CD14^+^CD206^+^ monocytes, and CD14^+^CD163^+^ monocytes. Additionally, the reduction of CD14^+^CD206^+^ monocytes and CD14^+^CD163^+^ monocytes in the peripheral blood of SLE patients showed significant heterogeneity.^[27]^ These findings indicate that different leukocyte subsets are involved in the disease progression of SLE to varying degrees. However, our subtype analysis found no evidence of causality between individual WBC subtypes and SLE, implicating the total WBC count as the operative factor. Analyses across major leukocyte subtypes all indicated point estimates in a protective direction, although with wide confidence intervals that included the null value. This pattern suggests a potential broad, nonspecific role of immune cell abundance in SLE pathogenesis, rather than an effect confined to a single cell lineage. Future studies with greater power are needed to confirm these associations and dissect their distinct mechanisms.

IL-10 is a pleiotropic anti-inflammatory cytokine produced by nearly all major immune cell types. IL-10 gene-deficient mice develop severe lupus.^[28]^Furthermore, in monocytes from SLE patients, clustered regularly interspaced short palindromic repeats-based interference with the regulation of this enhancer attenuated the production of disease-associated cytokines.^[29]^ Single-cell sequencing has revealed that immune-related genes Jun proto-oncogene, AP-1 transcription factor subunit, human leukocyte antigen A, and immune-related alternative splicing events interact to modulate T cell effector functions, driving pathogenic rewiring of T cell responses in SLE.^[30]^ These studies collectively provide systematic evidence supporting the biological plausibility of WBC subsets and immune molecules in SLE pathogenesis.

### 4.2. Limitations and methodological considerations

Although MR-Egger and MR-PRESSO indicated no significant pleiotropy, residual confounding via shared biological pathways cannot be excluded. For instance, single-nucleotide polymorphisms (SNPs) in *JAK-STAT* genes (e.g., rs3184504) influence both WBC count and cytokine signaling, potentially biasing causal estimates.^[31]^ Future studies should employ multivariable MR to disentangle the direct effects of WBCs on correlated immune traits.

Our study is limited by its reliance on European-ancestry genetic data. Notably, SLE prevalence and severity differ across ethnicities, with African Americans exhibiting higher neutrophil counts and more severe disease.^[32]^ SLE prevalence and severity vary across populations. Thus, our findings may not generalize to other groups and require validation in multi-ancestry cohorts.

### 4.3. Potential influence of racial/ethnic variation

While our study provides insights into the role of leukocyte subsets in SLE pathogenesis, several considerations regarding generalizability should be noted. First, our analysis was based on European-ancestry genome-wide association study (GWAS) data, whereas SLE exhibits disparities in prevalence, clinical manifestations, and severity across different racial/ethnic groups (e.g., African Americans have higher disease severity than whites).^[33]^ Second, a cross-ethnic GWAS study in 2020 (750,000 samples) identified 5552 loci related to blood cell traits, some of which had significantly different allele frequencies in different ancestral populations (such as East Asia, Europe, and Africa),^[34]^ which could influence the observed associations between immune cell profiles and SLE risk. Future studies in multi-ancestral cohorts are needed to determine whether our findings extend to non-European populations and to explore potential ancestry-specific effects. Furthermore, our study was based exclusively on European-ancestry genetic data. Baseline WBC distributions and allele frequencies differ across ancestries, and replication in non-European cohorts is therefore a prerequisite for any clinical extrapolation.

### 4.4. Clinical implications

From a clinical perspective, MR estimates represent lifelong genetic predisposition, not transient WBC fluctuations, and thus our findings do not support therapeutic WBC elevation. The observed signal likely reflects systemic immune-set-point regulation rather than a direct WBC-targeted effect. Management of leukopenia in SLE, whether therapy‑induced or disease‑related, should follow current guidelines, as these clinical scenarios are biologically distinct from the genetic susceptibility captured by MR. In practice, cytopenias arising from immunosuppressants or active disease neither contradict nor inform the genetic association reported here.

Two clinical distinctions merit emphasis. First, treatment-induced leukopenia, for example, from mycophenolate mofetil, azathioprine, or cyclophosphamide, does not contradict our MR findings because MR estimates capture lifelong genetic predisposition rather than acquired cytopenia. Second, leukopenia arising from active disease should be managed per standard guidelines; our results do not justify altering monitoring or treatment thresholds based on the genetic association observed.

## 5. Future directions

1. Cell-type-specific MR analyses: Integrate single-cell eQTL data to dissect the effects of specific leukocyte subsets (e.g., neutrophils and Tregs) on SLE risk.
2. Functional validation: Clustered regularly interspaced short palindromic repeats-edited mouse models were used to test whether modulating the WBC count alters lupus-like phenotypes.
3. Cross-ancestry studies: Replicate analyses of African, Asian, and Hispanic populations to assess genetic and environmental modifiers.
4. Integration with omics data: MR is combined with proteomics or metabolomics to identify intermediary pathways linking WBCs to SLE (e.g., complement activation).

Taken together, this study provides robust genetic evidence consistent with a causal protective effect of elevated WBC counts in SLE pathogenesis, independent of confounding and suggesting reverse causality. These findings underscore the importance of leukocyte homeostasis in autoimmune regulation and advocate for targeted therapies that modulate WBC dynamics.

## Acknowledgments

We gratefully acknowledge the contributions of the original GWAS studies and databases that made these data available for secondary analyses. We extend our thanks to Vuckovic, D for providing Blood Cell Consortium data (ieu-b-30) and to Hom G for providing the public data of systemic lupus erythematosus (ieu-a-815).

## Author contributions

**Conceptualization:** Yan-He Zhu.

**Formal analysis:** Li Zhang, Qin-Yu Yao, Jia Liu.

**Methodology:** Li Zhang, Jia Liu.

**Software:** Qin-Yu Yao, Zi-Xuan Dang.

**Writing – original draft:** Xin-Feng Wang.








