# Supplementary material for: Causal relationship between genetically predicted white blood cell count and systemic lupus erythematosus: A Mendelian randomization study
Source: Medicine (Baltimore). 2026 Sep 25;105(39):e50878. doi: 10.1097/MD.0000000000050878 (PMC13619244; doi:10.1097/MD.0000000000050878)
Supplement: Supplementary file 1 [file medi-105-e50878-s001.docx]

**Supplementary Table S1 MR results of Basophil, Monocyte, Lymphocyte, Eosinophil, Neutrophil counts on systemic lupus erythematosus**

| **id.exposure** | **id.outcome** | **outcome** | **exposure** | **method** | **nsnp** | **b** | **se** | **pval** | **lo_ci** | **up_ci** | **or** | **or_lci95** | **or_uci95** |  |
| --- | --- | --- | --- | --- | --- | --- | --- | --- | --- | --- | --- | --- | --- | --- |
|  |  |  |  |  |  |  |  |  |  |  |  |  |  |  |
| SOuiMS | dXzVQ3 | Systemic lupus erythematosus | neutrophil cell count | MR Egger | 100 | 0.326390581748632 | 1.3165990641641 | 0.804727136016901 | -2.254143584 | 2.90692474751027 | 1.3859565925193 | 0.1049633975864 | 18.3004334893651 |  |
| SOuiMS | dXzVQ3 | Systemic lupus erythematosus | neutrophil cell count | Weighted median | 100 | 0.0927544592236491 | 0.139787783838539 | 0.506986267137247 | -0.181229597 | 0.366738515547187 | 1.09719229674202 | 0.834243796749264 | 1.44302054234134 |  |
| SOuiMS | dXzVQ3 | Systemic lupus erythematosus | neutrophil cell count | Inverse variance weighted | 100 | -0.412527291 | 0.399851250374181 | 0.302211791418266 | -1.196235742 | 0.371181159708106 | 0.661975130464287 | 0.30233012129347 | 1.4494456307509 |  |
| SOuiMS | dXzVQ3 | Systemic lupus erythematosus | neutrophil cell count | Simple mode | 100 | -0.112537795 | 0.181256920444676 | 0.536109052927917 | -0.467801359 | 0.24272576945475 | 0.893563574577409 | 0.626377935835192 | 1.27471900929416 |  |
| SOuiMS | dXzVQ3 | Systemic lupus erythematosus | neutrophil cell count | Weighted mode | 100 | 0.00241802653520651 | 0.122847873872717 | 0.984335790980771 | -0.238363806 | 0.243199859325732 | 1.0024209523191 | 0.787915990179246 | 1.27532348394115 |  |
|  |  |  |  |  |  |  |  |  |  |  |  |  |  |  |
| PXCG1N | 3TKS5c | Systemic lupus erythematosus | eosinophil cell count | MR Egger | 71 | 0.042777082260894 | 0.821218495794844 | 0.958607675439851 | -1.566811169 | 1.65236533401879 | 1.04370520851125 | 0.208709662074047 | 5.21931065120996 |  |
| PXCG1N | 3TKS5c | Systemic lupus erythematosus | eosinophil cell count | Weighted median | 71 | -0.164677194 | 0.476615674815745 | 0.729708292906619 | -1.098843917 | 0.769489528271053 | 0.848167453132585 | 0.333256132828405 | 2.15866403552079 |  |
| PXCG1N | 3TKS5c | Systemic lupus erythematosus | eosinophil cell count | Inverse variance weighted | 71 | -0.243466359 | 0.372692235962124 | 0.51358620302948 | -0.973943142 | 0.487010423099196 | 0.783905846662263 | 0.377591203094275 | 1.62744357229597 |  |
| PXCG1N | 3TKS5c | Systemic lupus erythematosus | eosinophil cell count | Simple mode | 71 | 0.366993796178058 | 0.907893871410343 | 0.687279353909646 | -1.412478192 | 2.14646578414233 | 1.44338896455927 | 0.243538998350549 | 8.5545712067547 |  |
| PXCG1N | 3TKS5c | Systemic lupus erythematosus | eosinophil cell count | Weighted mode | 71 | -0.049882034 | 0.59503855514503 | 0.93343093774224 | -1.216157602 | 1.11639353416506 | 0.95134164392678 | 0.296366740912364 | 3.05382081904033 |  |
|  |  |  |  |  |  |  |  |  |  |  |  |  |  |  |
|  |  |  |  |  |  |  |  |  |  |  |  |  |  |  |
| FxLwab | tlIbHa | Systemic lupus erythematosus | basophil cell count | MR Egger | 35 | -2.105702314 | 2.12596316777611 | 0.329151832844866 | -6.272590123 | 2.06118549509083 | 0.121760130419447 | 0.00188733382096183 | 7.85527668454827 |  |
| FxLwab | tlIbHa | Systemic lupus erythematosus | basophil cell count | Weighted median | 35 | -1.357974255 | 0.826197013562523 | 0.100249636859757 | -2.977320401 | 0.26137189167458 | 0.257181233240692 | 0.0509291207823784 | 1.29871055527997 |  |
| FxLwab | tlIbHa | Systemic lupus erythematosus | basophil cell count | Inverse variance weighted | 35 | -0.944420944 | 0.742881340645566 | 0.203623907126666 | -2.400468372 | 0.511626483655718 | 0.388904703311478 | 0.0906754735186241 | 1.66800196777272 |  |
| FxLwab | tlIbHa | Systemic lupus erythematosus | basophil cell count | Simple mode | 35 | -1.496909032 | 1.59918582489225 | 0.355851691216944 | -4.631313249 | 1.63749518443146 | 0.223820915254429 | 0.00974195708124235 | 5.14227292191498 |  |
| FxLwab | tlIbHa | Systemic lupus erythematosus | basophil cell count | Weighted mode | 35 | -2.193087228 | 1.33847876380274 | 0.110541703388914 | -4.816505605 | 0.430331148746284 | 0.111571768852607 | 0.00809502498519186 | 1.53776666874666 |  |
|  |  |  |  |  |  |  |  |  |  |  |  |  |  |  |
|  |  |  |  |  |  |  |  |  |  |  |  |  |  |  |
| 6ndOQ0 | Ur4DJo | Systemic lupus erythematosus | monocyte cell count | MR Egger | 63 | -1.90079908 | 1.53210812341575 | 0.219488248492016 | -4.903731002 | 1.10213284172441 | 0.149449149644134 | 0.00741885161932796 | 3.01058027244625 |  |
| 6ndOQ0 | Ur4DJo | Systemic lupus erythematosus | monocyte cell count | Weighted median | 63 | -0.396387329 | 0.586407524546875 | 0.499066801039336 | -1.545746077 | 0.752971419215619 | 0.672746071473627 | 0.213152783493462 | 2.12329986625335 |  |
| 6ndOQ0 | Ur4DJo | Systemic lupus erythematosus | monocyte cell count | Inverse variance weighted | 63 | -0.389092391 | 0.600149648535737 | 0.516774368895919 | -1.565385702 | 0.787200920101758 | 0.677671656340375 | 0.209007383025931 | 2.19723756720178 |  |
| 6ndOQ0 | Ur4DJo | Systemic lupus erythematosus | monocyte cell count | Simple mode | 63 | -0.440033004 | 1.10598966689304 | 0.692097394222703 | -2.607772751 | 1.72770674332989 | 0.644015165797241 | 0.0736985060347793 | 5.62773326207074 |  |
| 6ndOQ0 | Ur4DJo | Systemic lupus erythematosus | monocyte cell count | Weighted mode | 63 | -0.750331731 | 0.910382005414171 | 0.412988613044613 | -2.534680462 | 1.03401699966828 | 0.472209880126811 | 0.0792870504781645 | 2.81234034491908 |  |
|  |  |  |  |  |  |  |  |  |  |  |  |  |  |  |
|  |  |  |  |  |  |  |  |  |  |  |  |  |  |  |
| C0JPQx | cIHktr | Systemic lupus erythematosus | lymphocyte cell count | MR Egger | 84 | -1.522129307 | 0.996789493255882 | 0.130601310476789 | -3.475836714 | 0.431578099865316 | 0.218246677681098 | 0.0309359384677199 | 1.53968538463892 |  |
| C0JPQx | cIHktr | Systemic lupus erythematosus | lymphocyte cell count | Weighted median | 84 | -0.915811288 | 0.500252398775921 | 0.0671455653663367 | -1.89630599 | 0.0646834132080593 | 0.400191823373122 | 0.150122148941412 | 1.06682122940571 |  |
| C0JPQx | cIHktr | Systemic lupus erythematosus | lymphocyte cell count | Inverse variance weighted | 84 | -0.773035172 | 0.438485516148072 | 0.0779061796728774 | -1.632466784 | 0.0863964393153344 | 0.461609874400484 | 0.195446853819386 | 1.09023845603032 |  |
| C0JPQx | cIHktr | Systemic lupus erythematosus | lymphocyte cell count | Simple mode | 84 | -0.859737163 | 1.00350025742701 | 0.394056354625077 | -2.826597668 | 1.1071233413129 | 0.423273319484678 | 0.0592139769517383 | 3.02564212387898 |  |
| C0JPQx | cIHktr | Systemic lupus erythematosus | lymphocyte cell count | Weighted mode | 84 | -1.014284329 | 0.597077212650335 | 0.0931122166053856 | -2.184555666 | 0.155987007613425 | 0.36266188350681 | 0.112527722443345 | 1.16881101734665 |  |
