# Supplementary material for: Causal relationship between genetically predicted white blood cell count and systemic lupus erythematosus: A Mendelian randomization study
Source: Medicine (Baltimore). 2026 Sep 25;105(39):e50878. doi: 10.1097/MD.0000000000050878 (PMC13619244; doi:10.1097/MD.0000000000050878)
Supplement: Supplementary file 2 [file medi-105-e50878-s003.docx]

Supplementary Table S2 Heterogeneity, pleiotropy and MRresult before and after outlier removal

Heterogeneity

| id.exposure | id.outcome | method | Q | Q_df | Q_pval |
| --- | --- | --- | --- | --- | --- |
| Before outlier removal | | | | | |
| aiUkUP | coYvNm | MR Egger | 142.759701816704 | 63 | 3.99373733127717e-08 |
| aiUkUP | coYvNm | Inverse variance weighted | 143.086515261843 | 64 | 5.55784989437902e-08 |
| After outlier removal | | | | | |
| uMEJ4l | tvcXa8 | MR Egger | 85.8794765375118 | 59 | 0.0127306960958304 |
| uMEJ4l | tvcXa8 | Inverse variance weighted | 86.7102992465257 | 60 | 0.0136379450803058 |

Pleiotropy

| id.exposure | id.outcome | | egger_intercept | | se | pval |
| --- | --- | --- | --- | --- | --- | --- |
| Before outlier removal | | | | | | |
| aiUkUP | coYvNm | | 0.00697661776490841 | | 0.0183707764886409 | 0.705395474592018 |
| After outlier removal | | | | | | |
| uMEJ4l | | tvcXa8 | | 0.0121092534962798 | 0.01602809397146 | 0.452953647187848 |

MRresult

| id.exposure | id.outcome | method | nSNP | b | se | pval | lo_ci | up_ci | or | or_lci95 | or_uci95 |
| --- | --- | --- | --- | --- | --- | --- | --- | --- | --- | --- | --- |
| Before outlier removal | | | | | | | | | | | |
| aiUkUP | coYvNm | MR Egger | 65 | -1.234 | 0.858 | 0.155 | -2.916 | 0.448 | 0.291 | 0.054 | 1.564 |
| aiUkUP | coYvNm | Weighted median | 65 | -1.121 | 0.495 | 0.024 | -2.098 | -0.144 | 0.326 | 0.123 | 0.866 |
| aiUkUP | coYvNm | Inverse variance weighted | 65 | -0.957 | 0.446 | 0.032 | -1.830 | -0.083 | 0.384 | 0.160 | 0.920 |
| aiUkUP | coYvNm | Simple mode | 65 | -0.060 | 0.940 | 0.950 | -1.902 | 1.783 | 0.942 | 0.149 | 5.949 |
| aiUkUP | coYvNm | Weighted mode | 65 | -0.750 | 0.564 | 0.188 | -1.854 | 0.355 | 0.473 | 0.157 | 1.427 |
| After outlier removal | | | | | | | | | | | |
| uMEJ4l | tvcXa8 | MR Egger | 61 | -1.391 | 0.820 | 0.095 | -3.00 | 0.216 | 0.249 | 0.050 | 1.242 |
| uMEJ4l | tvcXa8 | Weighted median | 61 | -1.257 | 0.556 | 0.024 | -2.347 | -0.167 | 0.284 | 0.096 | 0.846 |
| uMEJ4l | tvcXa8 | Inverse variance weighted | 61 | -0.851 | 0.402 | 0.034 | -1.639 | -0.063 | 0.427 | 0.194 | 0.939 |
| uMEJ4l | tvcXa8 | Simple mode | 61 | -0.369 | 0.971 | 0.705 | -2.272 | 1.534 | 0.691 | 0.103 | 4.635 |
| uMEJ4l | tvcXa8 | Weighted mode | 61 | -1.107 | 0.564 | 0.054 | -2.213 | -0.002 | 0.330 | 0.109 | 0.998 |
