# Supplementary material for: Causal relationship between genetically predicted white blood cell count and systemic lupus erythematosus: A Mendelian randomization study
Source: Medicine (Baltimore). 2026 Sep 25;105(39):e50878. doi: 10.1097/MD.0000000000050878 (PMC13619244; doi:10.1097/MD.0000000000050878)
Supplement: Supplementary file 3 [file medi-105-e50878-s004.docx]

Supplementary Table S3 Summary statistics of genetic variants (SNPs) associated with white blood cell count and their effects on systemic lupus erythematosus risk in Mendelian randomization analysis

| **SNP** | **Effect allele exposure** | **Other allele .exposure** | **Effect allele outcome** | **Other allele outcome** | **Beta exposure** | **Beta outcome** | **Eaf exposure** | **eaf.outcome** | **remove** | **palindromic** | **ambiguous** | **id.outcome** | **se.outcome** | **pval.outcome** | **outcome** | **Mr keep.outcome** | **Pval origin.outcome** | **Data source.outcome** | **se.exposure** | **exposure** | **mr_keep exposure** | **Pvalexposure** | **Pval origin.exposure** | **id.exposure** | **Data source.exposure** | **action** | **SNP index** | **mr_keep** | **Samplesize outcome** |
| --- | --- | --- | --- | --- | --- | --- | --- | --- | --- | --- | --- | --- | --- | --- | --- | --- | --- | --- | --- | --- | --- | --- | --- | --- | --- | --- | --- | --- | --- |
| rs1007938 | G | A | G | A | -0.011156 | -0.0314987 | 0.40493 | NA | FALSE | FALSE | FALSE | Hl4Gkj | 0.0470882 | 0.833981083 | SLE | TRUE | reported | textfile | 0.001884 | WBC count | TRUE | 3.19E-09 | inferred | AMU8x2 | textfile | 2 | 1 | TRUE | NA |
| rs10087240 | T | C | T | C | 0.019951 | 0.0148886 | 0.457631 | NA | FALSE | FALSE | FALSE | Hl4Gkj | 0.0464189 | 0.902746472 | SLE | TRUE | reported | textfile | 0.001855 | WBC count | TRUE | 5.60E-27 | inferred | AMU8x2 | textfile | 2 | 1 | TRUE | NA |
| rs10146962 | C | T | C | T | -0.015367 | -0.130151 | 0.338281 | NA | FALSE | FALSE | FALSE | Hl4Gkj | 0.04876 | 0.036441815 | SLE | TRUE | reported | textfile | 0.00197 | WBC count | TRUE | 6.17E-15 | inferred | AMU8x2 | textfile | 2 | 1 | TRUE | NA |
| rs10498635 | T | C | T | C | -0.031169 | -0.0880665 | 0.18322 | NA | FALSE | FALSE | FALSE | Hl4Gkj | 0.0605062 | 0.511579343 | SLE | TRUE | reported | textfile | 0.002388 | WBC count | TRUE | 6.16E-39 | inferred | AMU8x2 | textfile | 2 | 1 | TRUE | NA |
| rs10808139 | A | G | A | G | 0.017618 | -0.179605 | 0.118535 | NA | FALSE | FALSE | FALSE | Hl4Gkj | 0.07048 | 0.004608929 | SLE | TRUE | reported | textfile | 0.002845 | WBC count | TRUE | 5.92E-10 | inferred | AMU8x2 | textfile | 2 | 1 | TRUE | NA |
| rs10828725 | T | G | T | G | -0.049726 | -0.0148091 | 0.364782 | NA | FALSE | FALSE | FALSE | Hl4Gkj | 0.0496303 | 0.295588137 | SLE | TRUE | reported | textfile | 0.001914 | WBC count | TRUE | 8.30E-149 | inferred | AMU8x2 | textfile | 2 | 1 | TRUE | NA |
| rs10864368 | C | T | C | T | 0.021532 | 0.00471108 | 0.502821 | NA | FALSE | FALSE | FALSE | Hl4Gkj | 0.0460051 | 0.664339385 | SLE | TRUE | reported | textfile | 0.001838 | WBC count | TRUE | 1.07E-31 | inferred | AMU8x2 | textfile | 2 | 1 | TRUE | NA |
| rs10931934 | C | T | C | T | 0.01504 | 0.0423857 | 0.600163 | NA | FALSE | FALSE | FALSE | Hl4Gkj | 0.0465689 | 0.432059939 | SLE | TRUE | reported | textfile | 0.001882 | WBC count | TRUE | 1.33E-15 | inferred | AMU8x2 | textfile | 2 | 1 | TRUE | NA |
| rs10940473 | T | C | T | C | -0.013587 | -0.0407178 | 0.691742 | NA | FALSE | FALSE | FALSE | Hl4Gkj | 0.0500911 | 0.572544174 | SLE | TRUE | reported | textfile | 0.001996 | WBC count | TRUE | 9.96E-12 | inferred | AMU8x2 | textfile | 2 | 1 | TRUE | NA |
| rs11064881 | A | G | A | G | -0.032266 | 0.0372958 | 0.073548 | NA | FALSE | FALSE | FALSE | Hl4Gkj | 0.0849955 | 0.488486988 | SLE | TRUE | reported | textfile | 0.003547 | WBC count | TRUE | 9.31E-20 | inferred | AMU8x2 | textfile | 2 | 1 | TRUE | NA |
| rs11688303 | T | C | T | C | 0.021084 | -0.0137947 | 0.120295 | NA | FALSE | FALSE | FALSE | Hl4Gkj | 0.0667867 | 0.554069189 | SLE | TRUE | reported | textfile | 0.002892 | WBC count | TRUE | 3.09E-13 | inferred | AMU8x2 | textfile | 2 | 1 | TRUE | NA |
| rs12203592 | T | C | T | C | 0.022394 | -0.353395 | 0.210255 | NA | FALSE | FALSE | FALSE | Hl4Gkj | 0.0603033 | 0.031287438 | SLE | TRUE | reported | textfile | 0.002349 | WBC count | TRUE | 1.52E-21 | inferred | AMU8x2 | textfile | 2 | 1 | TRUE | NA |
| rs1260326 | C | T | C | T | -0.032863 | 0.057947 | 0.601256 | NA | FALSE | FALSE | FALSE | Hl4Gkj | 0.0467377 | 0.829322217 | SLE | TRUE | reported | textfile | 0.001875 | WBC count | TRUE | 8.93E-69 | inferred | AMU8x2 | textfile | 2 | 1 | TRUE | NA |
| rs12630592 | T | G | T | G | -0.012328 | 0.0610951 | 0.3731 | NA | FALSE | FALSE | FALSE | Hl4Gkj | 0.0471853 | 0.150863438 | SLE | TRUE | reported | textfile | 0.001992 | WBC count | TRUE | 6.06E-10 | inferred | AMU8x2 | textfile | 2 | 1 | TRUE | NA |
| rs1429216 | G | T | G | T | -0.00998 | 0.0718183 | 0.311709 | NA | FALSE | FALSE | FALSE | Hl4Gkj | 0.0498139 | 0.149758334 | SLE | TRUE | reported | textfile | 0.001998 | WBC count | TRUE | 5.88E-07 | inferred | AMU8x2 | textfile | 2 | 1 | TRUE | NA |
| rs1506609 | G | A | G | A | 0.021031 | 0.0223479 | 0.799464 | NA | FALSE | FALSE | FALSE | Hl4Gkj | 0.0573036 | 0.733081061 | SLE | TRUE | reported | textfile | 0.002326 | WBC count | TRUE | 1.54E-19 | inferred | AMU8x2 | textfile | 2 | 1 | TRUE | NA |
| rs1577452 | G | A | G | A | -0.009924 | -0.0459289 | 0.723067 | NA | FALSE | FALSE | FALSE | Hl4Gkj | 0.0512283 | 0.426146573 | SLE | TRUE | reported | textfile | 0.002081 | WBC count | TRUE | 1.85E-06 | inferred | AMU8x2 | textfile | 2 | 1 | TRUE | NA |
| rs1684618 | G | A | G | A | 0.008632 | 0.117321 | 0.511199 | NA | FALSE | FALSE | FALSE | Hl4Gkj | 0.0461806 | 0.061974067 | SLE | TRUE | reported | textfile | 0.001875 | WBC count | TRUE | 4.15E-06 | inferred | AMU8x2 | textfile | 2 | 1 | TRUE | NA |
| rs17257349 | A | G | A | G | 0.009849 | 0.0139029 | 0.339808 | NA | FALSE | FALSE | FALSE | Hl4Gkj | 0.0482646 | 0.381407299 | SLE | TRUE | reported | textfile | 0.001947 | WBC count | TRUE | 4.22E-07 | inferred | AMU8x2 | textfile | 2 | 1 | TRUE | NA |
| rs1727638 | A | G | A | G | 0.009317 | -0.00954541 | 0.44206 | NA | FALSE | FALSE | FALSE | Hl4Gkj | 0.0465294 | 0.84311361 | SLE | TRUE | reported | textfile | 0.001851 | WBC count | TRUE | 4.82E-07 | inferred | AMU8x2 | textfile | 2 | 1 | TRUE | NA |
| rs1738074 | C | T | C | T | -0.020403 | 0.112609 | 0.567796 | NA | FALSE | FALSE | FALSE | Hl4Gkj | 0.0468129 | 0.103685255 | SLE | TRUE | reported | textfile | 0.001853 | WBC count | TRUE | 3.39E-28 | inferred | AMU8x2 | textfile | 2 | 1 | TRUE | NA |
| rs175714 | C | T | C | T | 0.010451 | -0.0237165 | 0.577258 | NA | FALSE | FALSE | FALSE | Hl4Gkj | 0.0468743 | 0.491515253 | SLE | TRUE | reported | textfile | 0.001877 | WBC count | TRUE | 2.58E-08 | inferred | AMU8x2 | textfile | 2 | 1 | TRUE | NA |
| rs1788661 | T | C | T | C | -0.009625 | 0.0806579 | 0.541992 | NA | FALSE | FALSE | FALSE | Hl4Gkj | 0.0462185 | 0.114645761 | SLE | TRUE | reported | textfile | 0.001858 | WBC count | TRUE | 2.22E-07 | inferred | AMU8x2 | textfile | 2 | 1 | TRUE | NA |
| rs2076211 | T | C | T | C | -0.018018 | 0.0695261 | 0.158474 | NA | FALSE | FALSE | FALSE | Hl4Gkj | 0.0612334 | 0.192959871 | SLE | TRUE | reported | textfile | 0.002546 | WBC count | TRUE | 1.47E-12 | inferred | AMU8x2 | textfile | 2 | 1 | TRUE | NA |
| rs2082382 | A | G | A | G | -0.017857 | 0.0266419 | 0.55412 | NA | FALSE | FALSE | FALSE | Hl4Gkj | 0.0467373 | 0.302698313 | SLE | TRUE | reported | textfile | 0.00186 | WBC count | TRUE | 7.95E-22 | inferred | AMU8x2 | textfile | 2 | 1 | TRUE | NA |
| rs2180369 | C | T | C | T | 0.02298 | 0.0596439 | 0.110762 | NA | FALSE | FALSE | FALSE | Hl4Gkj | 0.0720906 | 0.557455237 | SLE | TRUE | reported | textfile | 0.003071 | WBC count | TRUE | 7.27E-14 | inferred | AMU8x2 | textfile | 2 | 1 | TRUE | NA |
| rs2241621 | C | A | C | A | -0.015164 | -0.0449734 | 0.587598 | NA | FALSE | FALSE | FALSE | Hl4Gkj | 0.0466442 | 0.106998823 | SLE | TRUE | reported | textfile | 0.001879 | WBC count | TRUE | 7.02E-16 | inferred | AMU8x2 | textfile | 2 | 1 | TRUE | NA |
| rs2290669 | C | A | C | A | -0.047616 | -0.131905 | 0.786444 | NA | FALSE | FALSE | FALSE | Hl4Gkj | 0.0575744 | 0.2214573 | SLE | TRUE | reported | textfile | 0.002308 | WBC count | TRUE | 1.45E-94 | inferred | AMU8x2 | textfile | 2 | 1 | TRUE | NA |
| rs2314339 | T | C | T | C | 0.027194 | -0.0991576 | 0.124894 | NA | FALSE | FALSE | FALSE | Hl4Gkj | 0.0681675 | 0.575524744 | SLE | TRUE | reported | textfile | 0.002851 | WBC count | TRUE | 1.45E-21 | inferred | AMU8x2 | textfile | 2 | 1 | TRUE | NA |
| rs2412771 | C | T | C | T | -0.018201 | -0.00796817 | 0.416739 | NA | FALSE | FALSE | FALSE | Hl4Gkj | 0.0467693 | 0.693666945 | SLE | TRUE | reported | textfile | 0.001883 | WBC count | TRUE | 4.21E-22 | inferred | AMU8x2 | textfile | 2 | 1 | TRUE | NA |
| rs2476601 | G | A | G | A | 0.052755 | -0.340749 | 0.899826 | NA | FALSE | FALSE | FALSE | Hl4Gkj | 0.0752219 | 0.006824801 | SLE | TRUE | reported | textfile | 0.003045 | WBC count | TRUE | 3.04E-67 | inferred | AMU8x2 | textfile | 2 | 1 | TRUE | NA |
| rs2839584 | T | C | T | C | -0.010449 | -0.00883895 | 0.784081 | NA | FALSE | FALSE | FALSE | Hl4Gkj | 0.055543 | 0.804520473 | SLE | TRUE | reported | textfile | 0.002243 | WBC count | TRUE | 3.19E-06 | inferred | AMU8x2 | textfile | 2 | 1 | TRUE | NA |
| rs2857633 | C | T | C | T | -0.013283 | 0.0488752 | 0.727142 | NA | FALSE | FALSE | FALSE | Hl4Gkj | 0.0532999 | 0.959668854 | SLE | TRUE | reported | textfile | 0.002098 | WBC count | TRUE | 2.43E-10 | inferred | AMU8x2 | textfile | 2 | 1 | TRUE | NA |
| rs301819 | G | A | G | A | -0.015802 | 0.174472 | 0.582558 | NA | FALSE | FALSE | FALSE | Hl4Gkj | 0.046616 | 0.273173772 | SLE | TRUE | reported | textfile | 0.00187 | WBC count | TRUE | 2.91E-17 | inferred | AMU8x2 | textfile | 2 | 1 | TRUE | NA |
| rs3184504 | C | T | C | T | -0.067191 | 0.0878481 | 0.517084 | NA | FALSE | FALSE | FALSE | Hl4Gkj | 0.0468971 | 0.066186589 | SLE | TRUE | reported | textfile | 0.001836 | WBC count | TRUE | 3.26E-293 | inferred | AMU8x2 | textfile | 2 | 1 | TRUE | NA |
| rs3732378 | A | G | A | G | 0.020274 | -0.0565704 | 0.172487 | NA | FALSE | FALSE | FALSE | Hl4Gkj | 0.0624782 | 0.473960337 | SLE | TRUE | reported | textfile | 0.00243 | WBC count | TRUE | 7.23E-17 | inferred | AMU8x2 | textfile | 2 | 1 | TRUE | NA |
| rs3747869 | C | A | C | A | 0.038842 | -0.0732505 | 0.900449 | NA | FALSE | FALSE | FALSE | Hl4Gkj | 0.0775344 | 0.80282798 | SLE | TRUE | reported | textfile | 0.00322 | WBC count | TRUE | 1.66E-33 | inferred | AMU8x2 | textfile | 2 | 1 | TRUE | NA |
| rs3773312 | A | G | A | G | 0.017874 | -0.00310482 | 0.134067 | NA | FALSE | FALSE | FALSE | Hl4Gkj | 0.0698567 | 0.862925296 | SLE | TRUE | reported | textfile | 0.00285 | WBC count | TRUE | 3.57E-10 | inferred | AMU8x2 | textfile | 2 | 1 | TRUE | NA |
| rs3847147 | A | G | A | G | -0.011891 | 0.0544882 | 0.306976 | NA | FALSE | FALSE | FALSE | Hl4Gkj | 0.0507106 | 0.603694195 | SLE | TRUE | reported | textfile | 0.002003 | WBC count | TRUE | 2.91E-09 | inferred | AMU8x2 | textfile | 2 | 1 | TRUE | NA |
| rs3956550 | C | A | C | A | 0.010083 | 0.0121738 | 0.245972 | NA | FALSE | FALSE | FALSE | Hl4Gkj | 0.0547307 | 0.736513989 | SLE | TRUE | reported | textfile | 0.002168 | WBC count | TRUE | 3.31E-06 | inferred | AMU8x2 | textfile | 2 | 1 | TRUE | NA |
| rs4707609 | C | T | C | T | -0.021092 | -0.064851 | 0.360123 | NA | FALSE | FALSE | FALSE | Hl4Gkj | 0.0483177 | 0.297211767 | SLE | TRUE | reported | textfile | 0.001914 | WBC count | TRUE | 3.07E-28 | inferred | AMU8x2 | textfile | 2 | 1 | TRUE | NA |
| rs4909945 | C | T | C | T | 0.011456 | 0.0216323 | 0.688902 | NA | FALSE | FALSE | FALSE | Hl4Gkj | 0.049756 | 0.387049936 | SLE | TRUE | reported | textfile | 0.001986 | WBC count | TRUE | 8.00E-09 | inferred | AMU8x2 | textfile | 2 | 1 | TRUE | NA |
| rs533483 | A | G | A | G | -0.017399 | 0.162969 | 0.243389 | NA | FALSE | FALSE | FALSE | Hl4Gkj | 0.0536302 | 0.031639528 | SLE | TRUE | reported | textfile | 0.002188 | WBC count | TRUE | 1.84E-15 | inferred | AMU8x2 | textfile | 2 | 1 | TRUE | NA |
| rs560194 | C | T | C | T | -0.013586 | -0.00995033 | 0.530014 | NA | FALSE | FALSE | FALSE | Hl4Gkj | 0.0461876 | 0.639689173 | SLE | TRUE | reported | textfile | 0.001843 | WBC count | TRUE | 1.68E-13 | inferred | AMU8x2 | textfile | 2 | 1 | TRUE | NA |
| rs571497 | A | G | A | G | -0.036357 | -0.0171462 | 0.154843 | NA | FALSE | FALSE | FALSE | Hl4Gkj | 0.0623489 | 0.767932415 | SLE | TRUE | reported | textfile | 0.002584 | WBC count | TRUE | 5.80E-45 | inferred | AMU8x2 | textfile | 2 | 1 | TRUE | NA |
| rs6045615 | C | A | C | A | -0.019319 | 0.0205089 | 0.330205 | NA | FALSE | FALSE | FALSE | Hl4Gkj | 0.0483362 | 0.952912886 | SLE | TRUE | reported | textfile | 0.001982 | WBC count | TRUE | 1.90E-22 | inferred | AMU8x2 | textfile | 2 | 1 | TRUE | NA |
| rs6115458 | G | A | G | A | 0.009125 | -0.0382587 | 0.3556 | NA | FALSE | FALSE | FALSE | Hl4Gkj | 0.0478916 | 0.437564419 | SLE | TRUE | reported | textfile | 0.001967 | WBC count | TRUE | 3.50E-06 | inferred | AMU8x2 | textfile | 2 | 1 | TRUE | NA |
| rs630505 | C | T | C | T | 0.012464 | 0.0383251 | 0.265394 | NA | FALSE | FALSE | FALSE | Hl4Gkj | 0.0513514 | 0.76685985 | SLE | TRUE | reported | textfile | 0.002102 | WBC count | TRUE | 3.04E-09 | inferred | AMU8x2 | textfile | 2 | 1 | TRUE | NA |
| rs6500550 | T | C | T | C | -0.02072 | 0.0009995 | 0.304495 | NA | FALSE | FALSE | FALSE | Hl4Gkj | 0.0499723 | 0.828015754 | SLE | TRUE | reported | textfile | 0.002036 | WBC count | TRUE | 2.52E-24 | inferred | AMU8x2 | textfile | 2 | 1 | TRUE | NA |
| rs6710055 | G | A | G | A | -0.01056 | -0.0276152 | 0.386192 | NA | FALSE | FALSE | FALSE | Hl4Gkj | 0.0469909 | 0.639362264 | SLE | TRUE | reported | textfile | 0.001992 | WBC count | TRUE | 1.15E-07 | inferred | AMU8x2 | textfile | 2 | 1 | TRUE | NA |
| rs6755895 | C | T | C | T | -0.023327 | 0.037079 | 0.231769 | NA | FALSE | FALSE | FALSE | Hl4Gkj | 0.0549115 | 0.775512853 | SLE | TRUE | reported | textfile | 0.002239 | WBC count | TRUE | 2.04E-25 | inferred | AMU8x2 | textfile | 2 | 1 | TRUE | NA |
| rs6866260 | G | A | G | A | -0.008924 | 0.025523 | 0.334778 | NA | FALSE | FALSE | FALSE | Hl4Gkj | 0.0490817 | 0.765880479 | SLE | TRUE | reported | textfile | 0.001951 | WBC count | TRUE | 4.78E-06 | inferred | AMU8x2 | textfile | 2 | 1 | TRUE | NA |
| rs696 | T | C | T | C | 0.015003 | -0.0349021 | 0.36576 | NA | FALSE | FALSE | FALSE | Hl4Gkj | 0.0483621 | 0.530454331 | SLE | TRUE | reported | textfile | 0.001912 | WBC count | TRUE | 4.27E-15 | inferred | AMU8x2 | textfile | 2 | 1 | TRUE | NA |
| rs6985508 | A | G | A | G | -0.026558 | 0.0217615 | 0.357998 | NA | FALSE | FALSE | FALSE | Hl4Gkj | 0.0485798 | 0.763285478 | SLE | TRUE | reported | textfile | 0.002018 | WBC count | TRUE | 1.48E-39 | inferred | AMU8x2 | textfile | 2 | 1 | TRUE | NA |
| rs7163368 | C | T | C | T | 0.011768 | 0.040822 | 0.212969 | NA | FALSE | FALSE | FALSE | Hl4Gkj | 0.0553201 | 0.889166516 | SLE | TRUE | reported | textfile | 0.002248 | WBC count | TRUE | 1.65E-07 | inferred | AMU8x2 | textfile | 2 | 1 | TRUE | NA |
| rs7541193 | T | G | T | G | 0.009705 | -0.040822 | 0.535503 | NA | FALSE | FALSE | FALSE | Hl4Gkj | 0.0466173 | 0.221040065 | SLE | TRUE | reported | textfile | 0.001859 | WBC count | TRUE | 1.78E-07 | inferred | AMU8x2 | textfile | 2 | 1 | TRUE | NA |
| rs755951 | C | A | C | A | -0.013467 | 0.016536 | 0.402765 | NA | FALSE | FALSE | FALSE | Hl4Gkj | 0.0472398 | 0.944638191 | SLE | TRUE | reported | textfile | 0.001888 | WBC count | TRUE | 9.82E-13 | inferred | AMU8x2 | textfile | 2 | 1 | TRUE | NA |
| rs7803075 | G | A | G | A | -0.02444 | 0.0259334 | 0.7342 | NA | FALSE | FALSE | FALSE | Hl4Gkj | 0.0510994 | 0.953297162 | SLE | TRUE | reported | textfile | 0.00211 | WBC count | TRUE | 5.03E-31 | inferred | AMU8x2 | textfile | 2 | 1 | TRUE | NA |
| rs7850247 | C | T | C | T | 0.01034 | -0.0620354 | 0.514717 | NA | FALSE | FALSE | FALSE | Hl4Gkj | 0.0464143 | 0.49764999 | SLE | TRUE | reported | textfile | 0.001861 | WBC count | TRUE | 2.76E-08 | inferred | AMU8x2 | textfile | 2 | 1 | TRUE | NA |
| rs7864482 | G | A | G | A | 0.012766 | 0.0246001 | 0.377314 | NA | FALSE | FALSE | FALSE | Hl4Gkj | 0.0475704 | 0.819210858 | SLE | TRUE | reported | textfile | 0.001902 | WBC count | TRUE | 1.92E-11 | inferred | AMU8x2 | textfile | 2 | 1 | TRUE | NA |
| rs7917772 | A | G | A | G | 0.016558 | 0.0227395 | 0.630809 | NA | FALSE | FALSE | FALSE | Hl4Gkj | 0.0481408 | 0.780572056 | SLE | TRUE | reported | textfile | 0.001904 | WBC count | TRUE | 3.42E-18 | inferred | AMU8x2 | textfile | 2 | 1 | TRUE | NA |
| rs8705 | A | G | A | G | -0.026397 | 0.0620354 | 0.316569 | NA | FALSE | FALSE | FALSE | Hl4Gkj | 0.0505723 | 0.892385284 | SLE | TRUE | reported | textfile | 0.002004 | WBC count | TRUE | 1.27E-39 | inferred | AMU8x2 | textfile | 2 | 1 | TRUE | NA |
| rs876383 | A | G | A | G | -0.010061 | 0.0440169 | 0.443368 | NA | FALSE | FALSE | FALSE | Hl4Gkj | 0.0465017 | 0.38104652 | SLE | TRUE | reported | textfile | 0.001895 | WBC count | TRUE | 1.10E-07 | inferred | AMU8x2 | textfile | 2 | 1 | TRUE | NA |
| rs9504961 | T | C | T | C | 0.010277 | 0.079735 | 0.683497 | NA | FALSE | FALSE | FALSE | Hl4Gkj | 0.0492884 | 0.11119647 | SLE | TRUE | reported | textfile | 0.001992 | WBC count | TRUE | 2.48E-07 | inferred | AMU8x2 | textfile | 2 | 1 | TRUE | NA |
| rs9526795 | C | T | C | T | -0.01411 | -0.0934903 | 0.258932 | NA | FALSE | FALSE | FALSE | Hl4Gkj | 0.0532882 | 0.166956372 | SLE | TRUE | reported | textfile | 0.002116 | WBC count | TRUE | 2.59E-11 | inferred | AMU8x2 | textfile | 2 | 1 | TRUE | NA |
